# Cost‐Effectiveness of NT‐proBNP–Guided Cardiovascular Screening in Chinese Adults With Type 2 Diabetes: Real‐World Evidence From the Hong Kong Diabetes Biobank

**DOI:** 10.1111/dom.70924

**Published:** 2026-06-03

**Authors:** Abby Q. Y. Li, Benjamin Yarnoff, Claudia H. T. Tam, Juliana C. N. Chan, Ronald C. W. Ma, Juliana N. M. Lui, Hong Kong Diabetes Biobank Study Group

**Affiliations:** ^1^ Department of Medicine and Therapeutics, Prince of Wales Hospital The Chinese University of Hong Kong Shatin Hong Kong; ^2^ Li Ka Shing Institute of Health Sciences, The Chinese University of Hong Kong, Prince of Wales Hospital Shatin Hong Kong; ^3^ Evidera Washington USA; ^4^ Hong Kong Institute of Diabetes and Obesity, Prince of Wales Hospital The Chinese University of Hong Kong Shatin Hong Kong; ^5^ Asia Diabetes Foundation Shatin Hong Kong

**Keywords:** cardiovascular disease, cost‐effectiveness, diabetes complications, heart failure, SGLT2 inhibitor, type 2 diabetes

## Abstract

**Aims:**

Heart failure (HF) is among the most costly and prevalent complications of type 2 diabetes (T2D), with annual incremental healthcare costs reaching US$12800 in Hong Kong. We evaluated the cost‐effectiveness of NT‐proBNP‐guided cardiovascular risk stratification to guide cardioprotective treatment.

**Materials and Methods:**

We developed a four‐state Markov cohort model with transition probabilities estimated from the Hong Kong Diabetes Biobank (2014–2019). Patients were classified as low or high risk of HF based on NT‐proBNP cut‐offs of 400 and 125 pg/mL. The analysis was conducted from a payer's perspective over a lifetime horizon, utilising annual cycles and 2023 costs. Outcomes included incremental cost‐effectiveness ratios (ICERs), life years (LY) and quality‐adjusted life years (QALY), with a willingness‐to‐pay (WTP) threshold of USD 50889 to USD 152667 (1–3 × Hong Kong GDP per capita).

**Results:**

NT‐proBNP screening utilising 400 pg/mL was cost‐saving (ICER: −USD $6558 per QALY) versus standard of care, yielding lower costs (−USD $846) and higher QALYs (0.129). The 125 pg/mL cut‐off was cost‐effective (ICER: USD $29 290 per QALY). Deterministic sensitivity analysis showed NT‐proBNP specificity as the key driver of ICERs in both cut‐offs. Probability Sensitivity Analysis results revealed a 100% probability of dominance for the 400 pg/mL cut‐off and 98% for the 125 pg/mL cut‐off at Hong Kong’s willingness‐to‐pay thresholds (USD$50,889–152,667).

**Conclusions:**

Using NT‐proBNP to prioritise use of cardioprotective treatment on top of standard of care to reduce the risk of progression to HF in Chinese patients with T2D is cost‐effective, supporting its integration into clinical practise.

**Abbreviations**
CV: Cardiovascular
DSA: deterministic sensitivity analysis
GDP: gross domestic product
HF: Heart failure
HKDB: Hong Kong Diabetes Biobank
ICER: incremental cost‐effectiveness ratio
LY: life years
NICE: National Institute for Health and Care Excellence
NT‐proBNP: N‐terminal pro‐B‐type natriuretic peptide
PSA: probabilistic sensitivity analysis
QALY: quality‐adjusted life years
SGLT2i: Sodium‐glucose co‐transporter‐2 inhibitors
T2D: type 2 diabetes
WTP: willingness‐to‐pay threshold

## 1 Introduction

Type‐2 diabetes (T2D) is a public health concern worldwide with rising prevalence [1]. T2D is associated with micro‐ and macrovascular complications including heart failure (HF), resulting in significant morbidity and healthcare costs. In 2019, an estimated 56 million people had HF worldwide [2]. Patients with T2D had a higher HF risk than patients without T2D [3, 4], with a prevalence of 9%–22% [5].

The increasing prevalence of T2D exacerbates the economic burden on healthcare systems globally with the projected costs escalating from USD$1.3 trillion in 2015 to USD$2.2–2.5 trillion by 2030 [6]. Cardiovascular (CV) complications including HF account for 20%–49% of T2D treatment costs worldwide [7]. Patients with T2D and CV complications incur USD$3418 to USD$9705 more per patient than those without [7]. The economic burden attributed to HF alone is estimated at USD$108 billion per annum [8]. In the United States, HF incurred an average annual healthcare expenditure of USD$28950, five times higher than those without HF [9]. In Hong Kong, the annual public direct medical cost for a 65‐year‐old Chinese patient with T2D without complications was USD$1521 which increased by two‐ to three‐fold in those with HF [10].

Given the economic burden of comorbid HF in patients with T2D, early identification of HF risk through biomarker screening is recommended. N‐terminal pro‐B‐type natriuretic peptide (NT‐proBNP) is a robust biomarker of cardiovascular risk, which can be used to identify high‐risk patients and guide cardioprotective treatment to reduce HF‐related events and downstream healthcare costs [11, 12, 13]. The PONTIAC study confirmed that NT‐proBNP is a valuable biomarker for identifying patients with T2D at higher risk of cardiac events [14]. Elevated NT‐proBNP was associated with higher risks of incident cardiovascular complications, with HRs ranging from 3.32 for cardiovascular disease to 4.46 for atrial fibrillation, and demonstrated strong discriminative capacity (C‐index 0.89 for congestive heart failure) [15].

Cost‐effectiveness analysis of NT‐proBNP screening specifically targeting patients with T2D has been conducted in Europeans [16], with paucity of data in Asian patients with T2D. Analysis in non‐Europeans is warranted given the inter‐ethnic differences in diabetes prevalence and incidence of CV complications [17]. Furthermore, Asians are three times more likely to develop HF than Europeans and have higher chronic kidney disease (CKD) risk, a major HF factor [18]. Considering East–West differences in disease epidemiology, behavioural factors, healthcare costs, and health service utilisation [17, 19, 20, 21], we aim to evaluate the cost‐effectiveness of NT‐proBNP screening to enable early identification and treatment of Hong Kong Chinese patients with T2D who are at high risk of developing HF.

## 2 Materials and Methods

### 2.1 Settings

The Hospital Authority (HA) serves over 90% of Hong Kong's population, predominantly Chinese, and governs all public hospitals [22]. Primary care for chronic disease management is mainly delivered through general outpatient clinics (GOPCs), while specialist care is provided at hospital‐based specialist outpatient clinics (SOPCs). The standard of care for HF detection involves referral to SOPCs for echocardiogram. In this study, we explored the cost‐effectiveness of implementing NT‐proBNP‐guided cardiovascular risk stratification in patients with T2D attending SOPCs to identify high‐risk patients for early initiation of cardioprotective treatment.

### 2.2 Study Design

A decision tree followed by a four‐state Markov cohort model—‘no HF’, ‘chronic HF’, ‘advanced HF’ and ‘dead’ (Figure S1) was developed to evaluate the cost‐effectiveness of NT‐proBNP‐guided cardioprotective treatment in T2D patients. The decision tree captured initial treatment decisions and short‐term outcomes, classifying patients as low or high risk for progression to chronic HF using a 400 pg/mL cut‐off for NT‐proBNP, as recommended by UK National Institute for Health and Care Excellence (NICE) [23]. This threshold is also adopted in Asia according to the joint guideline developed by the Japanese Circulation Society and Japanese Heart Failure Society [24]. Utilising a 400 pg/mL threshold for NT‐proBNP screening considers real‐world implementation, ensuring that clinical practises remain both effective and sustainable. When utilising a 125 pg/mL threshold, a significant proportion of patients (24.7%) were classified as ‘high risk’ in the HKDB cohort, raising concerns on the availability of resources and manpower for conducting subsequent echocardiograms for ‘high risk’ patients for HF [25]. The Markov model was used to simulate long‐term outcomes and disease progression. Patients entered the Markov model in a no HF state and might transition to chronic HF, advanced HF or death.

Patients with NT‐proBNP level higher than 400 pg/mL (high risk) were assumed to undergo echocardiogram examination for confirmation of cardiac dysfunction for initiation of SGLT2i cardioprotective drug treatment on top of standard of care. The latter includes renin‐angiotensin system inhibitors (RASi), beta‐blockers, and thiazide/thiazide‐like diuretics. We assume there will be no treatment carried out on low‐risk patients in this analysis (Figure S1). In this study, we simulated patients with T2D aged above 45 years (236 862 individuals) estimated from age‐ and sex‐specific data from the Census and Statistics Department of Hong Kong as of year‐end 2022 [26].

### 2.3 Study Population

Model parameters were derived from Chinese patients with T2D enrolled in the HKDB with retrospective measurement of NT‐proBNP in stored serum collected during structured assessment in 2014–2015 with censoring of outcomes using hospitalisation data on December 31, 2019. The HKDB is a multicentre cohort launched across 11 public Hong Kong hospitals for multiomic discovery [25]. The protocol adopted the Hong Kong Diabetes Register (HKDR) framework to assess risk factors and complications [27]. The study design, recruitment methods, baseline data collection and biochemical investigations of the HKDB have been described [25, 28]. All participants provided written informed consent. HF was identified using ICD‐9 primary diagnosis code (428). Chronic HF was defined as admission within 1 year during the entire follow‐up period and advanced HF as more than one HF admission within 1 year during the entire follow‐up period [29].

### 2.4 Model Clinical Parameters

Table S1 lists clinical parameters entered into the model for estimating transition probabilities. Transition probabilities between health states were derived from the HKDB cohort and existing literature [30], adjusted for age‐related risk [31]. Mortality rates for individuals without HF were extracted from Hong Kong life tables in 2023 [32] and obtained risk of mortality for T2D from literature [33]. Mortality rates for T2D patients with HF were estimated from 5‐year follow‐up data in the HKDB cohort for patients aged 50 and above, and proxied from Hong Kong age‐sex specific mortality rates [34] for patients aged 15–59. Advanced HF mortality rates were estimated by applying a hazard ratio (HR) of 5.0 to chronic HF mortality rates [30], and advanced HF incidence was further adjusted using age‐specific relatives (RR 1.09 for 70 years; RR 1.08 for 80 years) [31] to account for increased prevalence with age. All transition probabilities are applied by age rather than by time since model start; as TTE cohort ages through each annual cycle, the model applies the corresponding age‐ and sex‐specific rates, ensuring that the 55‐year time horizon reflects age‐related progression of risk. Treatment effects for standard of care (RASi and beta blockers) and SGLT2i were derived from the PONTIAC [14] and CANVAS studies [14, 35] respectively. The proportion of high‐risk patients receiving cardioprotective treatment was set at 80% based on clinical expert opinion, and treatment adherence was set at 91% [36]; both parameters were varied in the deterministic sensitivity analysis (DSA) to assess robustness. NT‐proBNP screening sensitivity (72.0%) and specificity (60.0%) values were derived from a European study predicting 10‐year all‐cause death [37].

### 2.5 Model Cost and Utility Parameters

The cost and utility parameters of the model are presented in Table S2. Costs were reported in 2023 US dollars. This analysis included costs for NT‐proBNP, standard of care, cardioprotective treatment (SGLT2i), and costs for chronic HF and advanced HF management [21, 38, 39, 40, 41]. Non‐entitled HA charges were applied to value healthcare resource use, as these more closely approximate the economic opportunity cost of services than heavily subsidized public‐sector fees (> 90%) for eligible residents. Baseline utilities were derived from age‐stratified EQ‐5D‐5L Hong Kong population norms [42], with HF‐ and T2D‐specific decrements reported in local and regional studies [43, 44].

### 2.6 Study Outcomes and Sensitivity Analyses

Outcomes included costs, life years (LYs), and quality‐adjusted life‐years (QALYs) gained. The incremental cost‐effectiveness ratio (ICER) compared SGLT2i cardioprotective treatment plus standard of care versus standard of care alone. The base case used an NT‐proBNP cutoff value of 400 pg/mL, while the 125 pg/mL cut‐off was evaluated in scenario analysis. In clinical trials, patients with NT‐proBNP levels ≥ 125 pg/mL had higher HF event rates than patients with lower value [45, 46].

We conducted DSA using lower and upper ranges of plus or minus 10% for each parameter, and probabilistic sensitivity analysis (PSA) with 1000 simulations to quantify the overall uncertainty in the model's output. Key treatment effects (hazard ratios for SGLT2 inhibitors and intensified standard care) were varied across their 95% CIs, with upper bounds representing conservative assumptions to reflect potential attenuation in lower‐risk real‐world populations. Cost‐effectiveness was evaluated against the Hong Kong willingness‐to‐pay (WTP) threshold of 1.0–3.0 times the Hong Kong gross domestic product (GDP) per capita (USD$50889 to USD$152667), with both DSA and PSA results interpreted relative to this threshold.

The analysis was conducted from a health care sector perspective over a life‐time horizon of 55 cycles. Health outcomes, measured in LYs, QALYs and all costs were discounted at an annual rate of 3.5% over the lifetime horizon, consistent with NICE methodological guidance [47, 48] and the commonly adopted rate in published cost‐effectiveness analyses [49, 50, 51, 52] in Hong Kong. All calculations and analyses were conducted using Excel software.

The study was approved by the Joint Chinese University of Hong Kong–New Territories East Cluster Clinical Research Ethics Committee (ref no: 2021.559).

## 3 Results

The analysis included 1889 patients from the HKDB cohort (mean ± SD age: 60.8 ± 10.9 years, 59.9% men, LDL‐C: 2.34 ± 0.78 mmol/L, HbA1c: 7.51% ± 1.43%, duration of diabetes: 11.1 ± 8.6 years) followed up for 5 years with an accrual of 49 HF events diagnosed by ICD codes. At baseline, 132 (7%) patients had high risk for HF using NT‐proBNP cut‐off of ≥ 400 pg/mL (Table 1). During the follow up period of 5 years (9445 patient‐years), the HF incidence was 0.00517 patient‐years over 9445 patient‐years. In the cost‐effectiveness analysis, using a NT‐proBNP cut‐off at 400 pg/mL to screen for patients at high risk of HF for initiation of SGLT2i cardioprotective treatment was cost‐saving compared with standard of care alone. The screening strategy incurred an average cost of USD$32987 per patient per year with a gain of 0.129 QALYs, compared with USD$33833 per patient per year for standard of care. This corresponded to an ICER of ‐USD$6558 per QALY gained, with an annual healthcare cost savings of USD$846 per patient (Table 2).

**TABLE 1:** Patient characteristics in the Hong Kong Diabetes Biobank (HKDB).

	Overall (*n*/mean)	*n* (%/SD)
NT‐proBNP (≥ 400 pg/mL) (%)	132	(7.0)
Male (%)	1132	(59.9)
Age (mean (SD)) years	60.8	(10.9)
Age groups years		
20–44	150	(7.9)
45–64	1120	(59.3)
65–74	520	(27.5)
≥ 75	194	(10.3)
Age of diagnosis (mean (SD)) years	49.6	(11.5)
Duration of diabetes (mean (SD)) years	11.1	(8.6)
Smoking (%)		
Current smokers	226	(12.0)
Ex‐smokers	408	(21.6)
Non smokers	1254	(66.4)
Insulin (%)	687	(36.8)
Blood pressure lowering drugs (%)	1425	(76.1)
Lipid‐lowering drugs (%)	1280	(68.2)
Glucose‐lowering drugs (%)	1613	(86.9)
BMI (mean (SD))	26.42	(4.57)
Waist (mean (SD))	91.9	(11.4)
High‐density lipoprotein cholesterol (mean (SD)) mmol/L	1.29	(0.39)
Low‐density lipoprotein cholesterol (mean (SD)) mmol/L	2.34	(0.78)
HbA1c (mean (SD)) %	7.51	(1.43)

Abbreviations: BMI, Body mass index; HbA1c, Haemoglobin A1c; NT‐proBNP, N‐terminal pro‐B‐type natriuretic peptide; SD, standard deviation.

**TABLE 2:** Cost‐effectiveness of NT‐proBNP screening (400 pg/mL cut‐off) for cardioprotective treatment in type 2 diabetes compared with standard care.

Cost description	NT‐proBNP	No risk assessment	Incremental caving
Diagnostic test^a^	$9	$0	$9
Cardioprotective treatment (SGLT2i)	$8963	$4018	$4945
Standard care	$9494	$9438	$57
Heart failure^b^	$14 520	$20 377	−$5857
Total costs	$32 987	$33 833	−$846
LYs	20.257	20.130	0.127
QALYs	17.624	17.495	0.129
ICER‐Cost per LY	—	—	−$6651 (dominates)
ICER‐Cost per QALY	—	—	−$6558 (dominates)

Abbreviations: ICER, Incremental cost effectiveness ratio; LY, life years; QALY, Quality adjusted life years.

^a^ All costs are presented as annualised values in USD$.

^b^ Echocardiogram costs are included in heart failure costs for both intervention (NT‐proBNP) and control arm.

In the scenario analysis using the 125 pg/mL cut‐off, patients who screened positive for high HF risk and initiated SGLT2i cardioprotective treatment gained 0.063 QALYs compared with standard of care, at an incremental cost of USD$1850 per patient. The ICER was USD$29290 per QALY gained (Table S3), which was within WTP threshold range of one to three times Hong Kong GDP per capita (USD 50889 to USD 152667), indicating that this screening strategy would be considered cost‐effective in Hong Kong.

### 3.1 Sensitivity Analysis

**FIGURE 1:**
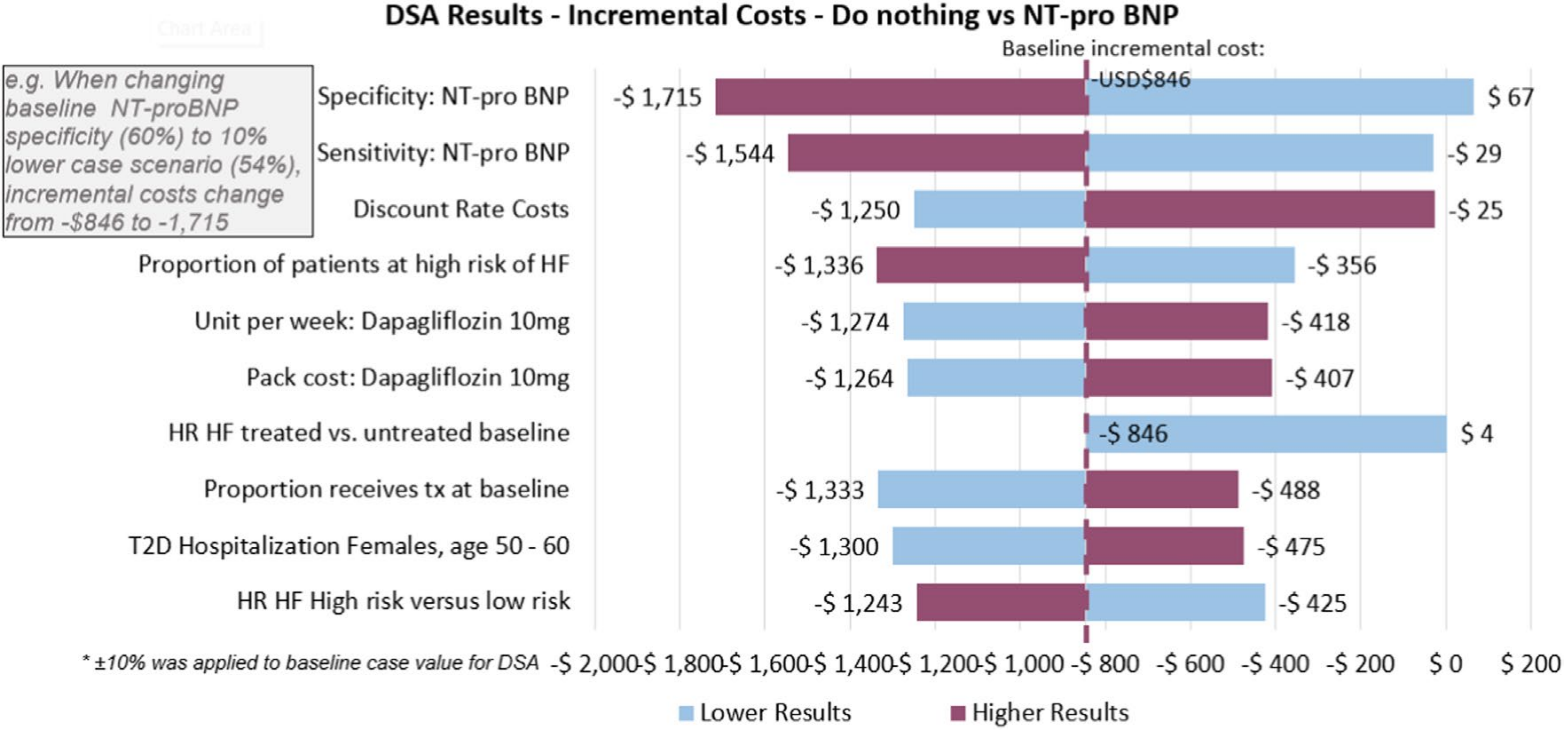
*Tornado plot of deterministic sensitivity analysis for incremental costs with 10 most impactful parameters*. The bars represent the variation in incremental costs when each input parameter is varied across its specified range. The vertical dotted line indicates the base‐case incremental cost. DSA, deterministic sensitivity analysis; HF, heart failure; HR, hazard ratio; NT‐proBNP, N‐terminal pro‐B‐type natriuretic peptide; T2D, type 2 diabetes.

**FIGURE 2:**
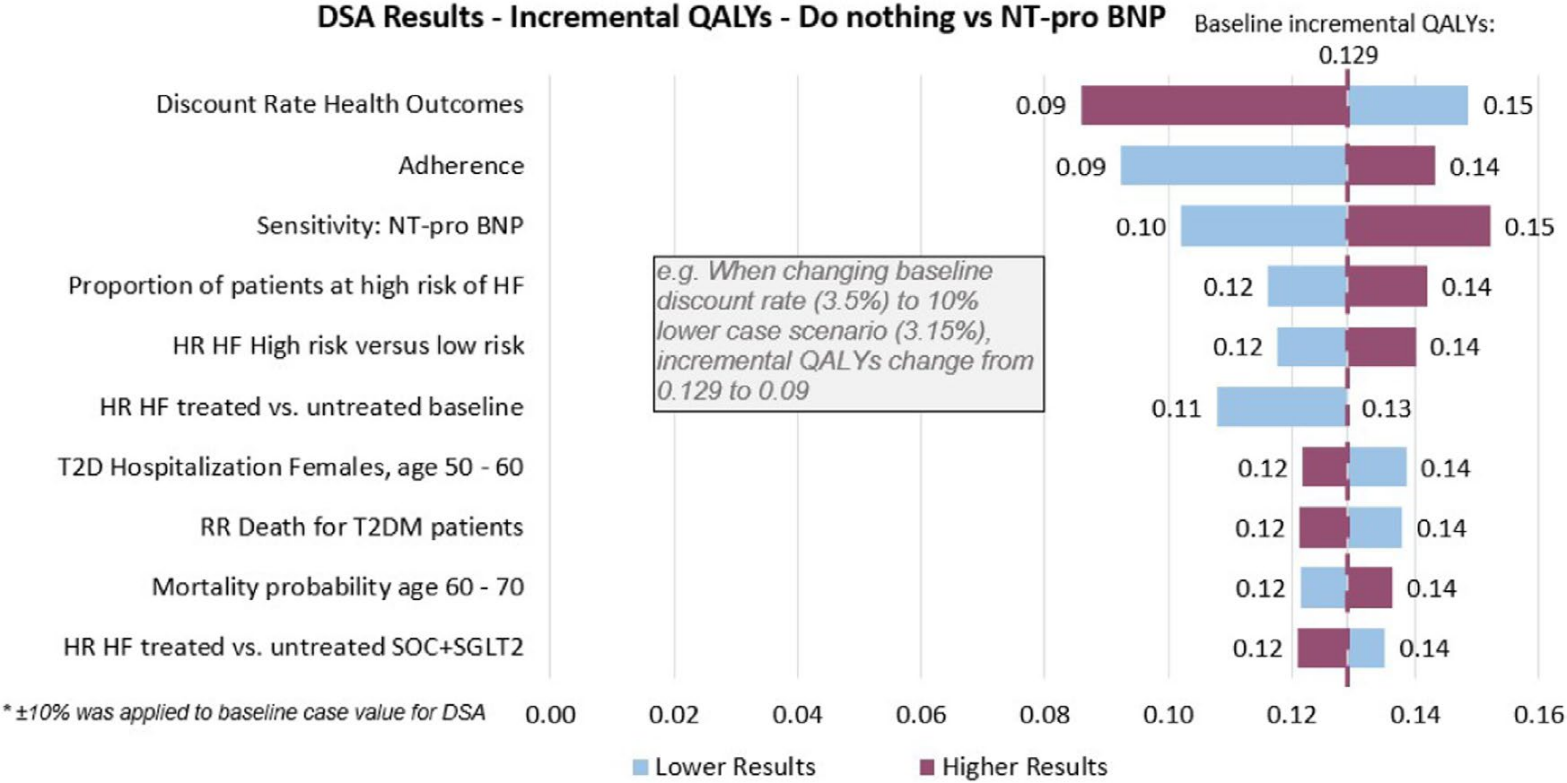
*Tornado plot of deterministic sensitivity analysis for incremental QALYs with 10 most impactful parameters*. The bars represent the variation in incremental QALYs when each input parameter is varied across its specified range. The vertical dotted line indicates the base‐case incremental QALYs. DSA, deterministic sensitivity analysis; HF, heart failure; HR, hazard ratio; NT‐proBNP, N‐terminal pro‐B‐type natriuretic peptide; QALY, quality‐adjusted life years; RR, relative risk; SGLT2, Sodium‐Glucose Co‐transporter‐2; SOC, standard of care; T2D, type 2 diabetes.

DSA for 400 pg/mL showed robust results, with findings consistent across plausible variations in parameter values for incremental costs and incremental QALYs (Figures 1 and 2). Parameters with greatest influence on incremental costs were NT‐proBNP specificity, NT‐proBNP sensitivity, cost discount rate, and proportion of patients at high risk of HF. QALY outcomes were most sensitive to health outcomes discount rate, drug adherence, NT‐proBNP sensitivity, and proportion of patients at high risk of HF.

In scenario analysis using a 125 pg/mL cut‐off, DSA results indicated that both incremental costs and incremental QALYs (Figure S2AB) were stable to plausible parameter variations. NT‐proBNP specificity was the primary influential factor on incremental costs, followed by proportion of high‐risk patients and HR of HF development among high‐risk and low‐risk patients. Similar to the 400 pg/mL cut‐off, the health outcomes discount rate remained the most significant parameter affecting QALYs.

**FIGURE 3:**
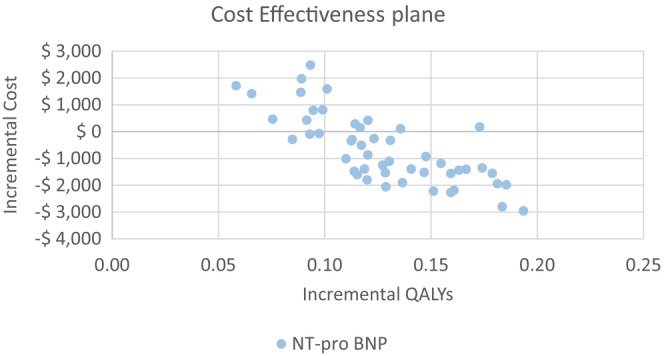
*Cost effectiveness plane of probabilistic sensitivity analysis*. Each point represents an iteration of the model, plotting the incremental costs against the incremental QALYs of NT‐proBNP screening compared to standard of care. NT‐proBNP, N‐terminal pro‐B‐type natriuretic peptide; QALY, quality‐adjusted life years.

**FIGURE 4:**
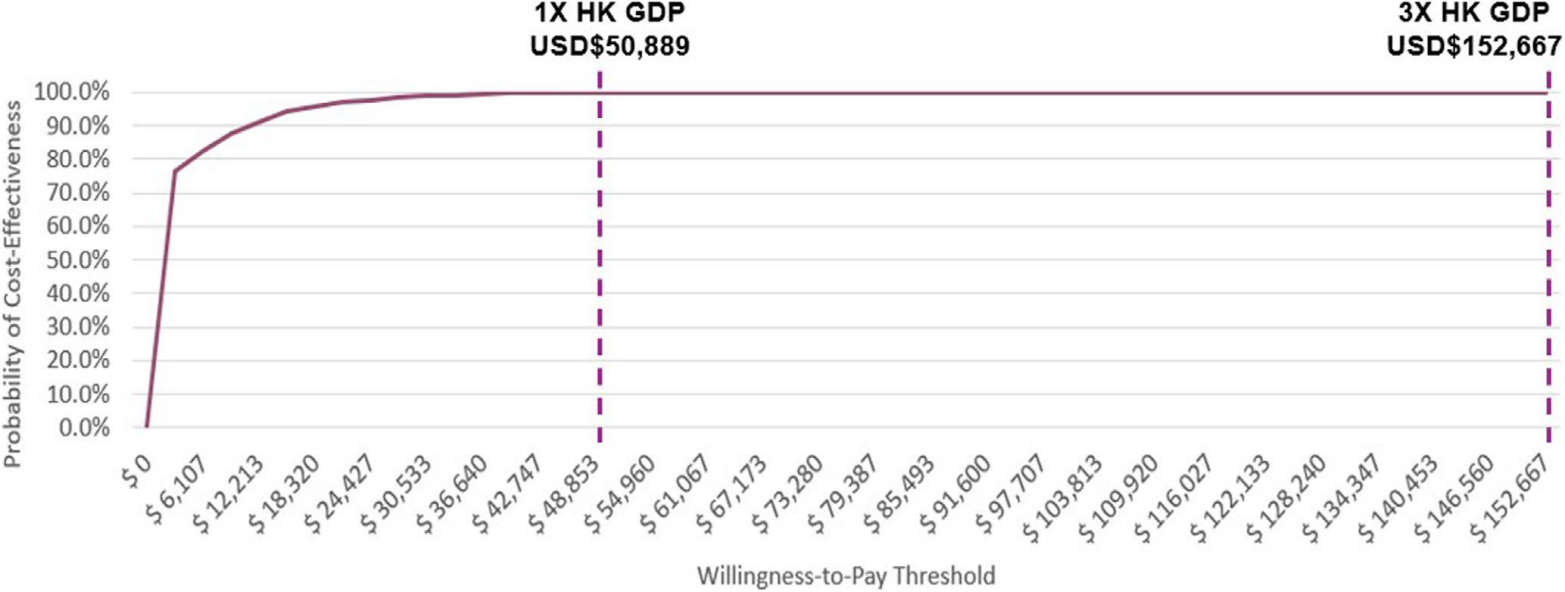
*Cost effectiveness acceptability curve of probabilistic sensitivity analysis*. The *y*‐axis represents the probability of cost‐effectiveness, while the *x*‐axis displays the varying threshold values. This curve demonstrates the probability that NT‐proBNP screening is cost‐effective across a range of potential willingness‐to‐pay thresholds. GDP, Gross domestic product; USD, US dollar.

In the PSA analysis, the 400 pg/mL NT‐proBNP cut‐off strategy was dominant in 70% of iterations (southeast quadrant: cost‐saving with greater effectiveness), with the remaining 30% cost‐effective within the WTP threshold range (Figure 3). The cost‐effectiveness acceptability curve indicated that all NT‐proBNP iterations (100%) were cost‐effective below the WTP threshold (Figure 4).

For the scenario analysis of NT‐proBNP cut‐off value of 125 pg/mL, PSA revealed all iterations clustered in the northeast or southeast quadrants of the cost‐effectiveness plane (Figure S3), with a 73%–98% probability of cost‐effectiveness within the WTP threshold range (Figure S4).

## 4 Discussion

In this study, we evaluated the long‐term impact and cost‐effectiveness of NT‐proBNP‐guided cardiovascular risk stratification to identify high‐risk patients and initiate SGLT2i cardioprotective treatment, compared with standard of care without biomarker screening in Chinese patients with T2D. Following NICE guidelines [23], we applied a 400 pg/mL NT‐proBNP cut‐off to distinguish low‐ and high‐risk patients for HF, with the latter group recommended for echocardiogram within 6 weeks followed by SGLT2i upon confirmation of cardiac dysfunction. Our analysis indicated that in Hong Kong Chinese patients with T2D aged ≥ 45 years, using NT‐proBNP cut‐off of 400 pg/mL to guide SGLT2i cardioprotective treatment was dominant with cost‐saving of −USD$6558 per QALY gained versus standard of care under the model assumptions. A 125 pg/mL cut‐off was also projected to be cost‐effective with USD$29290 per QALY, within the WTP threshold range. As previously discussed in the Bellicini study, NT‐proBNP is a biomarker of cardiovascular stress and elevations are not specific to HF, particularly in multimorbid populations where clinical and biomarker‐based criteria may encompass heterogeneous conditions rather than a single discrete entity [53]. Consistent with the Heart Failure Association of the ESC, which defined elevated NT‐proBNP in asymptomatic patients with cardiovascular risk factors as ‘heart stress’ rather than HF [13], our analysis utilised NT‐proBNP as a screening biomarker, further confirmed by echocardiogram, to identify patients with T2D at high risk of progression to HF for initiation of cardioprotective treatment, rather than to diagnose HF.

Our model‐based findings are broadly consistent with European economic evaluations suggesting that NT‐proBNP‐guided risk stratification may be cost‐effective. The prevalence of diabetes is threefold higher in Asians than Europeans, with different complication patterns influenced by factors such as genetics, lifestyle and healthcare access [17, 19, 20, 21]. While Southeast Asians have a higher prevalence of HF than Europeans, the ageing phenomenon and propensity for CKD in East Asia increase the HF burden [16, 17, 18]. These demographic and ethnic differences call for context‐relevant cost‐effectiveness analyses to inform practise and policies. To the best of our knowledge, this is the first cost‐effectiveness analysis of NT‐proBNP‐guided cardiovascular risk stratification to guide SGLT2i cardioprotective treatment in Asian patients with T2D. In Western settings, the cost‐effectiveness of NT‐proBNP diagnosis has been evaluated across different healthcare settings, such as emergency rooms and acute medical wards, or in patient groups such as elderly or mixed patient groups with or without T2D [16, 54, 55, 56]. Most published studies on NT‐proBNP have evaluated its role in diagnosis. In contrast, few studies have evaluated NT‐proBNP‐guided risk stratification. A study demonstrated that NT‐proBNP‐guided risk stratification is cost‐effective for asymptomatic left ventricular dysfunction [57], and our study suggested that NT‐proBNP‐guided risk stratification is not only cost‐saving but yields greater savings compared with NT‐proBNP diagnostic strategies.

Our cohort enrolled ambulatory patients with T2D attending medical clinics in hospital settings. Compared to in‐patient setting or high‐risk populations such as those with advance age or prior HF or CV complications, these patients had relatively low risk of HF at baseline, 7% of the study population (1889 participants) were screened high‐risk. Among these patients, 21 (15.9%) developed HF. Instead of using echocardiogram to diagnose cardiac dysfunction, we used ICD code to diagnose HF. None of our patients were on SGLT2i, and assuming initiation of SGLT2 in high‐risk patients, the 400 pg/cut‐ was cost‐saving with a gain of 0.127LYs and 0.129QALYs, with a reduction of USD $846 per patient. These projected ICERs were comparable to those reported in model‐based analyses conducted in Austria and Switzerland among patients with T2D [16] with ICERs of USD$3432 per QALY and $7051 per QALY, respectively. All these values fall within their local WTP thresholds [16].

PSA showed 100% probability of cost‐effectiveness for the 400 pg/mL cut‐off and 73% to 98% for the 125 pg/mL cut‐off within the WTP range. These findings aligned with other reports indicating NT‐proBNP‐guided intensive management was more effective and less costly than usual care [58]. While one study comparing five different screening strategies did not favour the 125 pg/mL cut‐off [59], these variations might reflect different settings and populations. In Chinese patients with T2D attending hospital‐based clinics, our scenario analysis using the European Society of Cardiology (ESC) recommended 125 pg/mL cut‐off for slow‐onset HF remained cost‐effective [60].

The cost‐effectiveness of NT‐proBNP in patients with T2D and comorbid HF significantly impacts costs and mortality. The abnormal internal milieu in patients with T2D increases risk for multiple complications, resulting in high medical costs primarily driven by hospitalizations and outpatient care services [10, 61]. In Hong Kong, CV complications including coronary heart disease, stroke, and HF were the top diabetes‐related complications [62].

Hospitalizations due to CV events ranked among top healthcare costs in patients with T2D. Among adults aged ≥ 75 years, HF accounted for the highest proportion of complication events. In a study conducted in 2016, patients aged ≥ 75 years with T2D accounted for 61.9% of all HF events in men and 83.3% of all HF events in women [62]. Older patients require more complex care with longer recovery periods and higher resource utilization. Their HF propensity contributes substantially to costs, which persist in subsequent years [21]. In Hong Kong, chronic heart failure ranked among top five complications in healthcare costs, both in the year of event and in subsequent years [21]. Hong Kong, as with other healthcare systems worldwide, faces growing financial constraints in healthcare expenditures, emphasising the need for early identification of high‐risk HF patients and early initiation of cardioprotective treatment to reduce healthcare economic burden.

NT‐proBNP‐guided risk stratification for SGLT2i cardioprotective treatment initiation illustrates the potential value of precision medicine. The EMPA‐REG OUTCOME, CANVAS, and DECLARE‐TIMI 58 trials confirmed that SGLT2i reduced CV events and hospitalizations or death due to HF in patients with prior CV events [35, 63, 64]. In real‐world setting, the majority of patients with T2D did not have CV complications and the cost‐effectiveness of SGLT2i in low‐risk patients for HF remained to be proven [62]. In this analysis, we included the cost for HF (including costs of echocardiogram, hospitalizations, follow‐ups and drugs) and diabetes using the HA costs for non‐entitled persons as well as costs for NT‐proBNP and SGLT2i. In an estimated 236 862 patients with T2D aged ≥ 45 attending hospital‐based clinics, using 400 pg/mL as a cut‐off value for initiation of SGLT2i use of a 400 pg/mL cut‐off for SGLT2i initiation was projected to result in 12 011 fewer HF cases over the 55‐year time horizon, corresponding to an estimated cost reduction of USD$846 per person and projected lifetime savings of USD$10.2 million. Given similar patterns of CV complications in Asia, our findings are relevant to other Asian populations which account for 50% of the global population with diabetes [19].

Our study has several strengths. This is the first cost‐effectiveness analysis of NT‐proBNP‐guided risk stratification in an Asian population using real‐world data, hospitalisation costs and quality of life measurements. Patients from HKDB were representative of those attending hospital‐based clinics in a public setting. Most clinical inputs were either calculated directly from HKDB data or adopted from local sources, which increased contextual relevance. Apart from the 400 pg/mL cut‐off adopted by NICE [23], we also evaluated the 125 pg/mL cut‐offs recommended by other professional organisations [65]. The framework of our model is adaptable and applicable to other countries and regions in Asia.

Outcome data were derived from our electronic medical records database with structured data fields, where echocardiographic imaging parameters (e.g., ejection fraction) [30] and unstructured clinical notes (e.g., NYHA functional class) were not available in this dataset. Thus, we utilised ICD codes and HF hospitalisations to classify HF as supported by validated HF categorisation [29]. This approach relies on clinical and biomarker‐based criteria rather than hemodynamic confirmation, and in multimorbid populations such as patients with T2D, conditions other than true cardiac congestion may be captured as HF. This is a common limitation of studies using administrative health data and would apply to both arms of the model equally. As HKDB patients were referred to diabetes centres in hospital‐based clinics for structured assessments, they likely had more risk factors and higher HF rates than primary care patients, limiting generalizability to low‐risk community clinic populations. The treatment effect estimates were derived from clinical trials (CANVAS and PONTIAC), which enrolled patients at higher cardiovascular risk than the HKDB cohort; applying trial‐derived relative risk reductions to a lower‐risk population may overestimate the absolute treatment benefit. However, the relative reduction in HF hospitalization with SGLT2i has been shown to be consistent across trials enrolling populations of varying cardiovascular risk—ranging from 26% to 35% across EMPA‐REG OUTCOME, DECLARE‐TIMI58, DAPA‐HF and EMPEROR‐reduced, and so on [66, 67]. To account for potential attenuation in real‐world settings, the upper bounds of the 95% confidence intervals of hazard ratios were tested in the DSA and the results remained robust. The model assumed immediate treatment initiation upon NT‐proBNP screening, whereas in real‐world practise, treatment initiation may be delayed due to clinical workflow, patient scheduling, or system‐level barriers. Delayed initiation were not explicitly modelled and could attenuate the magnitude and duration of treatment benefit, potentially reducing the cost effectiveness of the screening strategy. Future studies incorporating real world data on uptake rates, delayed initiation and adherence patterns would further enhance the generalizability of the findings. We only included direct healthcare costs, adding indirect costs such as productivity loss and caregiver burden would likely demonstrate even greater screening benefits.

## 5 Conclusion

In this model‐based analysis of patients with T2D attending hospital‐based clinics, NT‐proBNP‐guided risk stratification was projected to be cost‐effective across different cut‐offs (125 and 400 pg/mL) compared to standard of care. Sensitivity analyses suggested that these findings were generally robust to parameter variation within the tested ranges. These projections support the potential economic value of NT‐proBNP‐guided cardiovascular risk stratification to inform SGLT2i initiation. Prospective implementation studies would be required to confirm real‐world clinical and economic impact.

## Funding

This work was supported by the Roche Diagnostics (Hong Kong) Limited.

## Disclosure

Investigator‐initiated grants from Roche Diagnostics (Hong Kong) supported the NT‐proBNP measurements and the cost‐effectiveness analysis.

## Conflicts of Interest

The authors declare no conflicts of interest.

## Supporting information


**Figure S1:** Decision tree and Markov health state model for evaluating the cost‐effectiveness of NT‐proBNP‐guided cardioprotective treatment in patients with T2D.
**Table S1:** Model inputs on clinical parameters for transition probabilities.
**Table S2:** Model inputs on cost and quality of life decrements.
**Table S3:** Cost‐effectiveness results of NT‐proBNP screening (125 pg/mL cut‐off).
**Figure S2A:** Tornado plot of deterministic sensitivity analysis for NT‐proBNP screening (125 pg/mL cut‐off) for incremental costs with 10 most impactful parameters.
**Figure S2B:** Tornado plot of deterministic sensitivity analysis for NT‐proBNP screening (125 pg/mL cut‐off) for incremental QALYs with 10 most impactful parameters.
**Figure S3:** Cost effectiveness plane of probabilistic sensitivity analysis for NT‐proBNP screening (125 pg/mL cut‐off).
**Figure S4:** Cost effectiveness acceptability curve of probabilistic sensitivity analysis for NT‐proBNP screening (125 pg/mL cut‐off).

## Data Availability

The data supporting the findings of this study are not publicly available due to the inclusion of confidential patient information.
