# Supplementary material for: Cost‐Effectiveness of NT‐proBNP–Guided Cardiovascular Screening in Chinese Adults With Type 2 Diabetes: Real‐World Evidence From the Hong Kong Diabetes Biobank
Source: Diabetes Obes Metab. 2026 Jun 3;28(8):7342–51. doi: 10.1111/dom.70924 (PMC13341330; doi:10.1111/dom.70924)
Supplement: Supplementary file 1 — Figure S1: Decision tree and Markov health state model for evaluating the cost‐effectiveness of NT‐proBNP‐guided cardioprotective treatment in patients with T2D. Table S1: Model inputs on clinical parameters for transition probabilities. Table S2: Model inputs on cost and quality of life decrements. Table S3: Cost‐effectiveness results of NT‐proBNP screening (125 pg/mL cut‐off). Figure S2A: Tornado plot of deterministic sensitivity analysis for NT‐proBNP screening (125 pg/mL cut‐off) for incremental costs with 10 most impactful parameters. Figure S2B: Tornado plot of deterministic sensitivity analysis for NT‐proBNP screening (125 pg/mL cut‐off) for incremental QALYs with 10 most impactful parameters. Figure S3: Cost effectiveness plane of probabilistic sensitivity analysis for NT‐proBNP screening (125 pg/mL cut‐off). Figure S4: Cost effectiveness acceptability curve of probabilistic sensitivity analysis for NT‐proBNP screening (125 pg/mL cut‐off). [file DOM-28-7342-s001.docx]

**Supplementary Materials for**

**Cost‑Effectiveness of NT‑proBNP–Guided Cardiovascular Screening in Chinese Adults with Type 2 Diabetes: Real‑World Evidence from the Hong Kong Diabetes Biobank**

Abby Q.Y. Li^1,2^, Benjamin Yarnoff^3^, Claudia H.T. Tam^1,2^, Juliana C.N. Chan^1,2,4,5^, Ronald C.W. Ma^1,2,4^, Juliana N.M. Lui^1,2,4,5^ and Hong Kong Diabetes Biobank Study Group

^1^ Department of Medicine and Therapeutics, Prince of Wales Hospital, The Chinese University of Hong Kong, Shatin, Hong Kong.

^2^ Li Ka Shing Institute of Health Sciences, The Chinese University of Hong Kong, Prince of Wales Hospital, Shatin, Hong Kong.

^3^ Evidera, 7101 Wisconsin Ave., Suite 1400, Bethesda, Washington, USA.

^4^ Hong Kong Institute of Diabetes and Obesity, Prince of Wales Hospital, The Chinese University of Hong Kong, Shatin, Hong Kong.

^5^ Asia Diabetes Foundation, Shatin, Hong Kong.

**Table of Contents**

[Figure 1. Decision tree and Markov health state model for evaluating the cost-effectiveness of NT-pro BNP screening and cardioprotective follow-up treatment in patients with T2D 3](#_Toc230873590)

[Table 1. Model inputs on clinical parameters for transition probabilities 4](#_Toc230873591)

[Table 2. Model inputs on cost and quality of life decrements 6](#_Toc230873592)

[Table 3. Cost-effectiveness results for NT-proBNP screening (125 pg/mL cut-off) 8](#_Toc230873593)

[Figure 2(A). Tornado plot of deterministic sensitivity analysis for NT-proBNP screening (125 pg/mL cut-off) for incremental costs with 10 most impactful parameters 9](#_Toc230873594)

[Figure 2(B). Tornado plot of deterministic sensitivity analysis for NT-proBNP screening (125 pg/mL cut-off) for incremental QALYs with 10 most impactful parameters 9](#_Toc230873595)

[Figure 3. Cost Effectiveness plane of probabilistic sensitivity analysis for NT-proBNP screening (125 pg/mL cut-off) 10](#_Toc230873596)

[Figure 4. Cost effectiveness acceptability curve of probabilistic sensitivity analysis for NT-proBNP screening (125 pg/mL cut-off) 11](#_Toc230873597)

[References 12](#_Toc230873598)


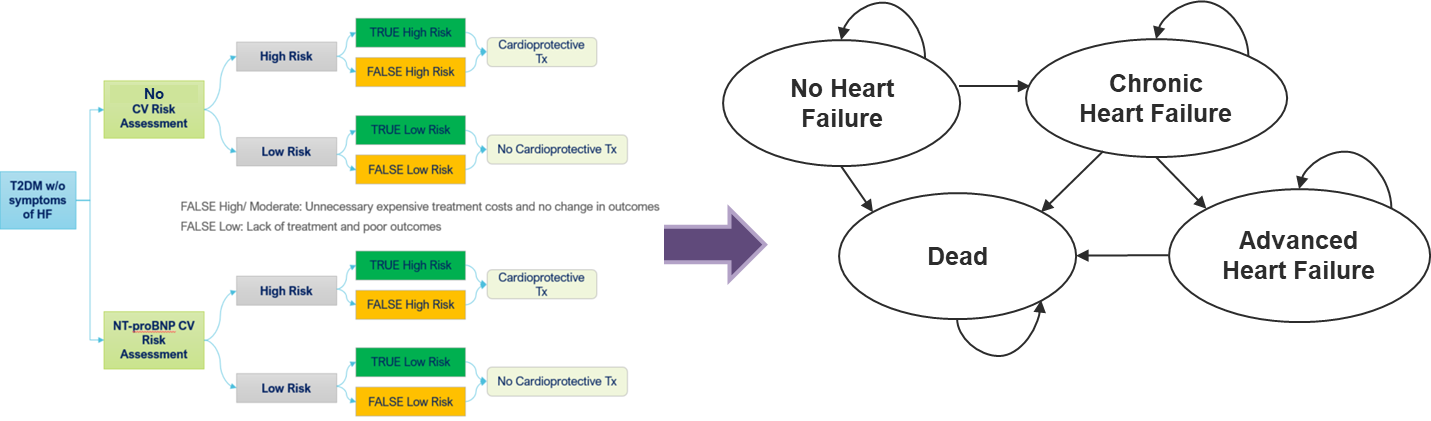


# Figure 1. Decision tree and Markov health state model for evaluating the cost-effectiveness of NT-proBNP–guided cardiovascular screening and cardioprotective follow-up treatment in patients with T2D

# Table 1. Model inputs on clinical parameters for transition probabilities

| **Model parameters** | **Base case value** | **Low value** | **High value** | **Distribution** | **Source** |
| --- | --- | --- | --- | --- | --- |
| **Settings** | | | | | |
| % Prevalence of T2DM | 0.850 | 0.769 | 0.935 | Beta | Hong Kong Centre for Health Protection^1^ |
| % Males | 0.456 | 0.412 | 0.501 | Beta | Census and Statistics Department of Hong Kong 2023^2^ |
| **Clinical inputs** | | | | | |
| NT-pro BNP sensitivity | 0.720 | 0.569 | 0.849 | Beta | Prausmuller et al. 2021^3^ |
| NT-pro BNP specificity | 0.60 | 0.480 | 0.714 | Beta | Prausmuller et al. 2021^3^ |
| Proportion of HF events over 10 years for low-risk patients | 0.00189 | 0.00170 | 0.00208 | Beta | Hong Kong Diabetes Biobank (HKDB) cohort data |
| Hazard ratio for HF (High risk vs low risk) | 4.00 | 3.60 | 4.40 | Log Normal | HKDB cohort data |
| Cumulative incidence of advanced HF over 6 years | 0.1150 | 0.110 | 0.130 | Beta | Subramanyam et al. |
| Risk ratio for advanced HF incidence (70 years) | 1.09 | 1.07 | 1.11 | Log Normal | Dunlay et al. 2021^4^ |
| Risk ratio for advanced HF incidence (80 years) | 1.08 | 1.06 | 1.09 | Log Normal | Dunlay et al. 2021^4^ |
| Risk ratio of death for patients with type-2 diabetes (T2D) | 1.59 | 1.43 | 1.75 | Log Normal | Wu et al. 2020^5^ |
| 1 Year mortality probability for patients with chronic HF with T2D | Age-stratified risk | Age-stratified risk | Age-stratified risk | Beta | HKDB cohort data |
| All-cause mortality in Hong Kong population | Age-stratified risk | Age-stratified risk | Age-stratified risk | Beta | Census and Statistics Department of Hong Kong Life tables 2023^2^ |
| Hazard ratio for all cause death (advanced vs chronic HF) | 5.00 | 4.50 | 5.60 | Log Normal | Subramaniam et al. 2022^6^ |
| HR HF hospitalization (treated vs. untreated) SGLT2i + SoC | 0.09 | 0.05 | 0.16 | Log Normal | Pontiac and CANVAS Program study |
| Proportion of patients at high risk of HF | 0.248 | 0.2232 | 0.2728 | Beta | HKDB cohort data |
| Treatment adherence | 90.8% | 67.0% | 100.0% | Beta | Leung 2014^7^ |

# Table 2. Model inputs on cost and quality of life decrements

| **Model parameters** | **Base case value** | **Low value** | **High value** | **Distribution** | **Source** |
| --- | --- | --- | --- | --- | --- |
| **Utility** | | | | | |
| Quality of life of Hong Kong general population | Age-stratified | Age-stratified | Age-stratified | Beta | Wong 2019^8^ |
| No HF (T2DM) | 0.86 | 0.65 | 0.98 | Beta | Lui 2023^9^  McMurray et al. 2018^10^  Trueman et al 2017^11^ |
| Chronic HF (T2DM) | 0.80 | 0.62 | 0.93 | Beta |  |
| Advanced HF (T2DM) | 0.76 | 0.60 | 0.89 | Beta |  |
| **Costs** | | | | | |
| NT-pro BNP | 9.20 | 8.30 | 10.20 | Gamma | Internal data |
| Diabetes management | 461.50 | 417.40 | 507.90 | Gamma | JADE diabetes risk assessment^12^ |
| HF treatment (one-off) | 13429.70 | 12145.70 | 14777.30 | Gamma | HA gazette^13^  CUHK Medical Centre pricing for HA patients^14^  Lui 2024^15^  Butler 2020^16^ |
| Chronic HF management (annual) | 17304.60 | 15650.10 | 19041.00 | Gamma |  |
| Advanced HF (one-off) | 52966.30 | 47902.20 | 58281.20 | Gamma |  |
| Advanced HF (annual) | 41357.40 | 37403.20 | 45507.30 | Gamma |  |
| Drug treatments |  |  |  |  |  |
| ARBs - Candesartan 8mg | 102.67 | 82.13 | 123.20 | Gamma | Price - Online catalogue from Lok Sin Tong Community Pharmacy ^17^  Percentage of cohort on treatment – HKDB cohort data |
| ARBs- Exforge 10mg/160mg | 233.33 | 186.67 | 280.00 | Gamma |  |
| ARBs- Exforge 5mg/160mg | 186.67 | 149.33 | 224.00 | Gamma |  |
| ARBs - Losartan 100mg | 140.00 | 112.00 | 168.00 | Gamma |  |
| ARNI- Sacubitril/Valsartan | 1466.67 | 1173.33 | 1760.00 | Gamma |  |
| MRAs - Spironolactone 25mg | 112.00 | 89.60 | 134.40 | Gamma |  |
| Beta blockers - Atenolol 50mg | 53.33 | 42.67 | 64.00 | Gamma |  |
| Beta blockers - Propranolol 10mg | 1400.00 | 1120.00 | 1680.00 | Gamma |  |
| SGLT2i - Dapagliflozin 10mg | 633.33 | 506.67 | 760.00 | Gamma |  |
| CCB - Amlodipine 5mg | 73.33 | 58.67 | 88.00 | Gamma |  |
| Diuretic - Hydrochlorothiazide 25 mg | 168.00 | 134.40 | 201.60 | Gamma |  |
| Diuretic - Indapamide 1.5mg | 168.00 | 134.40 | 201.60 | Gamma |  |
| Diuretic - Furosemide 40mg | 80.00 | 64.00 | 96.00 | Gamma |  |

ARB, Angiotensin II Receptor Blockers; ARNI, Angiotensin Receptor-Neprilysin Inhibitor; MRA, Mineralocorticoid receptor antagonists; SGLT2i, Sodium-glucose Cotransporter-2 Inhibitors; CCB, Calcium Channel Blockers; HKDB, Hong Kong Diabetes Biobank; JADE, Joint Asia Diabetes Evaluation JADE Program; T2D, type-2 diabetes; HA, Hospital Authority; CUHK, The Chinese University of Hong Kong.

# Table 3. Cost-effectiveness results for NT-proBNP screening (125 pg/mL cut-off)

| **Cost description*** | **NT-pro BNP** | **No risk assessment** | **Incremental** |
| --- | --- | --- | --- |
| Diagnostic costs* | $9 | $0 | $9 |
| Cardioprotective treatment (SGLT2i) | $9,093 | $4,123 | $4,970 |
| Standard care | $9,540 | $9,513 | $27 |
| Heart failure^†^ | $9,380 | $12,537 | -$3,157 |
| Total costs | $28,023 | $26,173 | $1,850 |
| LYs | 20.360 | 20.299 | 0.061 |
| QALYs | 17.730 | 17.666 | 0.063 |
| ICER-Cost per LY | .. | .. | $1,850  (More effective, more costly) |
| ICER-Cost per QALY | .. | .. | $29,290  (More effective, more costly) |

*All dollars are in USD.

^†^Echocardiogram costs are included in heart failure costs for both intervention (NT-proBNP) and control arm

LY, life years; ICER, Incremental cost effectiveness ratio; QALY, Quality adjusted life years; SGLT2i, Sodium-glucose Cotransporter-2 Inhibitors


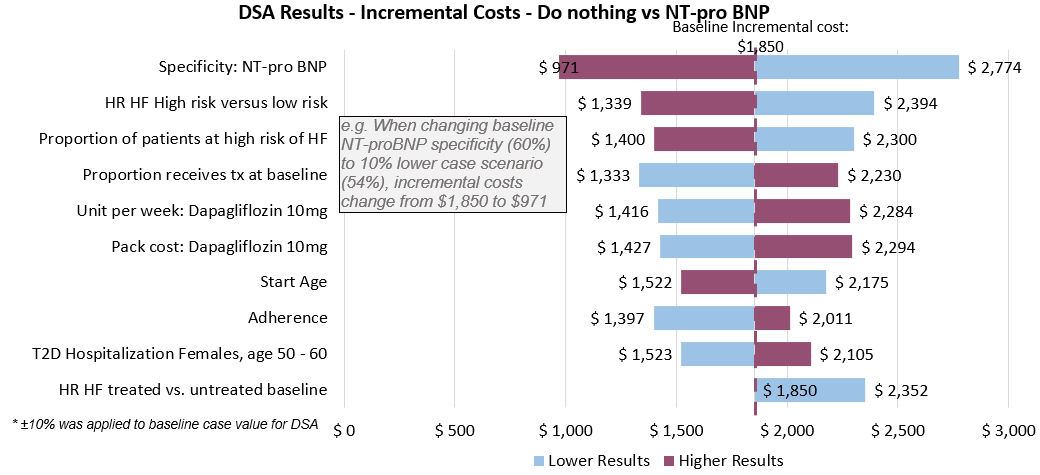


# Figure 2(A). Tornado plot of deterministic sensitivity analysis for NT-proBNP screening (125 pg/mL cut-off) for incremental costs with 10 most impactful parameters

The bars represent the variation in incremental costs when each input parameter is varied across its specified range. The vertical dotted line indicates the base-case incremental cost. DSA, deterministic sensitivity analysis; HF, heart failure; HR, hazard ratio; NT-proBNP, N-terminal pro-B-type natriuretic peptide; T2D, type 2 diabetes.


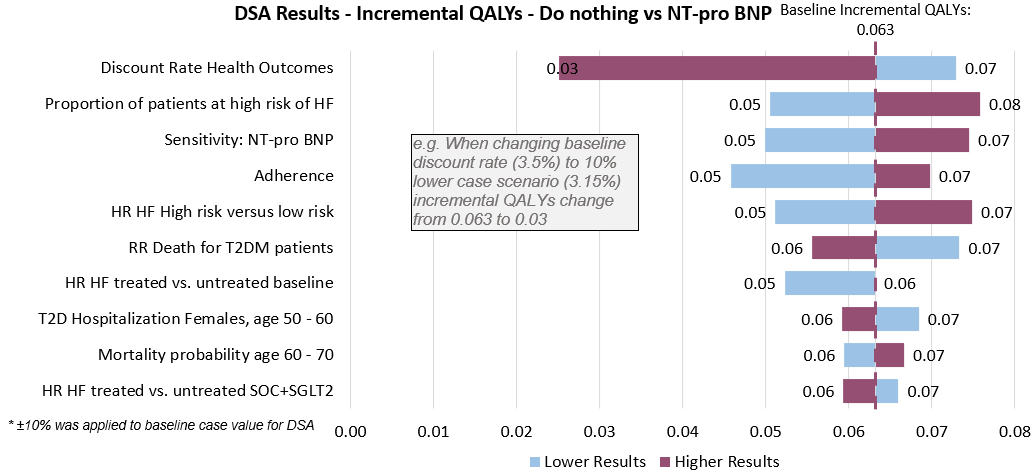


Figure 2(B). Tornado plot of deterministic sensitivity analysis for NT-proBNP screening (125 pg/mL cut-off) for incremental QALYs with 10 most impactful parameters

The bars represent the variation in incremental QALYs when each input parameter is varied across its specified range. The vertical dotted line indicates the base-case incremental QALYs. DSA, deterministic sensitivity analysis; HF, heart failure; HR, hazard ratio; NT-proBNP, N-terminal pro-B-type natriuretic peptide; QALY, quality-adjusted life years; RR, relative risk; SGLT2, Sodium-Glucose Co-transporter-2; SOC, standard of care; T2D, type 2 diabetes.

# Figure 3. Cost Effectiveness plane of probabilistic sensitivity analysis for NT-proBNP screening (125 pg/mL cut-off)

Each point represents an iteration of the model, plotting the incremental costs against the incremental QALYs of NT-proBNP screening compared to standard of care. NT-proBNP, N-terminal pro-B-type natriuretic peptide; QALY, quality-adjusted life years.

#


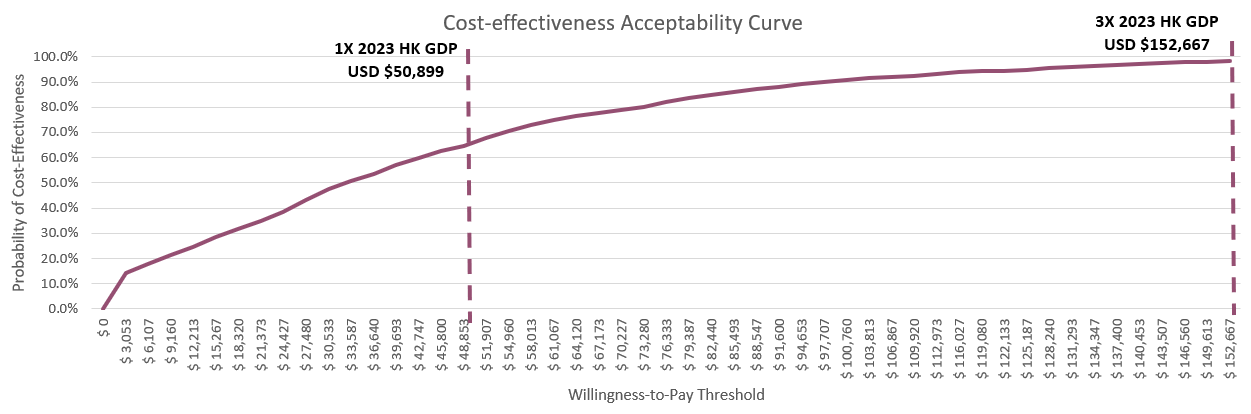


# **Figure 4. Cost effectiveness acceptability curve of probabilistic sensitivity analysis for** NT-proBNP screening **(125 pg/mL cut-off)**

The y-axis represents the probability of cost-effectiveness, while the x-axis displays the varying threshold values. This curve demonstrates the probability that NT-proBNP screening is cost-effective across a range of potential willingness-to-pay thresholds. GDP, Gross domestic product; USD, US dollar.

# References

1. Centre for Health Protection. Situation of Diabetes in Hong Kong.

2. The Census and Statistics Department (C&SD). Hong Kong Life Tables 2016-2046 Hong Kong2024 [cited 2025 6 June]. Available from: <https://www.censtatd.gov.hk/en/page_34.html>.

3. Prausmüller S, Resl M, Arfsten H, Spinka G, Wurm R, Neuhold S, et al. Performance of the recommended ESC/EASD cardiovascular risk stratification model in comparison to SCORE and NT-proBNP as a single biomarker for risk prediction in type 2 diabetes mellitus. Cardiovasc Diabetol. 2021;20(1):34.

4. Dunlay SM, Roger VL, Killian JM, Weston SA, Schulte PJ, Subramaniam AV, et al. Advanced Heart Failure Epidemiology and Outcomes: A Population-Based Study. JACC Heart Fail. 2021;9(10):722-32.

5. Wu H, Lau ESH, Ma RCW, Kong APS, Wild SH, Goggins W, et al. Secular trends in all-cause and cause-specific mortality rates in people with diabetes in Hong Kong, 2001-2016: a retrospective cohort study. Diabetologia. 2020;63(4):757-66.

6. Subramaniam AV, Weston SA, Killian JM, Schulte PJ, Roger VL, Redfield MM, et al. Development of Advanced Heart Failure: A Population-Based Study. Circ Heart Fail. 2022;15(5):e009218.

7. Leung DY, Bai X, Leung AY, Liu BC, Chi I. Prevalence of medication adherence and its associated factors among community-dwelling Chinese older adults in Hong Kong. Geriatrics & Gerontology International. 2015;15(6):789-96.

8. Wong EL, Cheung AW, Wong AY, Xu RH, Ramos-Goñi JM, Rivero-Arias O. Normative Profile of Health-Related Quality of Life for Hong Kong General Population Using Preference-Based Instrument EQ-5D-5L. Value Health. 2019;22(8):916-24.

9. Lui JNM, Lau ESH, Yang A, Wu H, Fu A, Lau V, et al. Temporal associations of diabetes-related complications with health-related quality of life decrements in Chinese patients with type 2 diabetes: A prospective study among 19 322 adults-Joint Asia Diabetes Evaluation (JADE) register (2007-2018). J Diabetes. 2024;16(6):e13503.

10. McMurray JJV, Trueman D, Hancock E, Cowie MR, Briggs A, Taylor M, et al. Cost-effectiveness of sacubitril/valsartan in the treatment of heart failure with reduced ejection fraction. Heart. 2018;104(12):1006-13.

11. Trueman D, Kapetanakis V, Briggs A, Lewis E, Rouleau J, Solomon SD, et al. P3373Better health-related quality of life in patients treated with sacubitril/valsartan compared with enalapril, irrespective of NYHA class: Analysis of EQ-5D in PARADIGM-HF. European Heart Journal. 2017;38(suppl_1).

12. Hong Kong Institute of Diabetes and Obesity. The Chinese University of Hong Kong Yao Chung Kit Diabetes Assessment Centre Hong Kong2023 [cited 2023 11 October]. Available from: <https://www.hkido.cuhk.edu.hk/Centres/CUHK-Yao-Chung-Kit-Diabetes-Assessment-Centre>.

13. Hospital Authority. Hospital Authority - Fees and Charges 2023 [cited 2023 28 October]. Available from: <https://www.ha.org.hk/visitor/ha_visitor_index.asp?Content_ID=10045&Lang=ENG>.

14. The Chinese University of Hong Kong Medical Centre. Cardiology Centre - Cardiac Services and Fees for Patients with Referral Letter issued by the Hospital Authority 2023 [cited 2023 28 September]. Available from: <https://www.cuhkmc.hk/medical-centres-allied-health/cardiology-centre>.

15. Lui JNM, Lau ESH, Li AQY, Zhang Y, Lim L-L, Chun Kwun O, et al. Temporal incremental healthcare costs associated with complications in Hong Kong Chinese patients with type 2 diabetes: A prospective study in Joint Asia diabetes evaluation (JADE) Register (2007&#x2013;2019). Diabetes Research and Clinical Practice. 2025;219.

16. Butler J, Djatche LM, Sawhney B, Chakladar S, Yang L, Brady JE, et al. Clinical and Economic Burden of Chronic Heart Failure and Reduced Ejection Fraction Following a Worsening Heart Failure Event. Adv Ther. 2020;37(9):4015-32.

17. Lok Sin Tong Community Pharmacy Services. Lok Sin Tong Community Pharmacy Services - product catalogue Hong Kong2023 [cited 2023 18 September]. Available from: <https://lstcps.hk/zh-hant/shopping/>.
